# The association between the metabolic score for insulin resistance (METS-IR) index and urinary incontinence in the United States: results from the National Health and Nutrition Examination Survey (NHANES) 2001–2018

**DOI:** 10.1186/s13098-023-01226-3

**Published:** 2023-12-02

**Authors:** Shangqi Cao, Linghao Meng, Lede Lin, Xu Hu, Xiang Li

**Affiliations:** Institute of Urology, Department of Urology, West China Medical School, West China Hospital, Sichuan University, 37 Guoxue Street, Chengdu, 610041 China

**Keywords:** The metabolic score for insulin resistance index, METS-IR index, Insulin resistance, Urinary incontinence, National Health and Nutrition Examination Survey, NHANES

## Abstract

**Background:**

The association between insulin resistance and urinary incontinence (UI) has not been investigated widely. The purpose of this study is to assess the relationship between a novel indicator for assessing insulin resistance the metabolic score for insulin resistance (METS-IR) index and urinary incontinence (UI).

**Methods:**

This study utilized data from National Health and Nutrition Examination Survey (NHANES) 2001–2018. Weighted multivariable logistic regression models were conducted to explore the association of METS-IR index with three types of UI [stress UI (SUI), urgency UI (UUI), and mixed UI (MUI)]. Smooth curve fitting was utilized to investigate the linear relationship. Subgroup analysis was used to examine the stability of the connection between METS-IR index and UI in different stratifications.

**Results:**

A total of 17,474 participants were included in this study, of whom 23.76% had SUI, 20.05% had UUI, and 9.59% had MUI. METS-IR index was positively associated with three types of UI with full adjustment [SUI: odds ratio (OR) = 1.023, 95% confidence interval (CI) 1.019–1.027; UUI: OR = 1.015, 95% CI 1.011–1.019; MUI: OR = 1.020, 95% CI 1.016–1.025, all p < 0.001]. After transferring METS-IR index into a categorical variable by quartiles, the positive connection between METS-IR index and UI was still observed in the highest METS-IR group compared to the lowest METS-IR interval (SUI: OR = 2.266, 95% CI 1.947–2.637, p < 0.001; UUI: OR = 1.534, 95% CI 1.344–1.750, p < 0.001; MUI: OR = 2.044, 95% CI 1.707–2.448, p < 0.001). The analysis of smooth curves fitting showed that METS-IR index was positively linearly related to three types of UI. Moreover, the association between METS-IR index and SUI was more significant in females compared to males (p for interaction < 0.05).

**Conclusion:**

An elevated METS-IR index was related to increased risks of three types of UI (SUI, UUI, and MUI) in the United States population. METS-IR index was more significantly connected to SUI in females than males. The association between insulin resistance and UI needs to be explored with more studies.

## Introduction

Urinary incontinence (UI) is a highly common problem defined as a complaint of involuntary loss of urine [1]. While it affects both sexes, it is notably more prevalent among the female population [2]. A longitudinal population-based survey in Sweden demonstrated that the incidences of UI were 21% in women and the prevalence of UI increased markedly from 1991 to 2007 [3]. A Korean EPIC study showed that 2.9% of males and 28.4% of females reported UI. The most common types of UI were stress UI in women and other UI in men [4]. A prospective study reported that there was a significant increase in the prevalence of UI from 4.5% in 1992 to 10.5% in 2003 in Sweden males [5]. The main classification of UI includes stress urinary incontinence (SUI), urgency urinary incontinence (UUI), and mixed urinary incontinence (MUI) [6]. According to the International Continence Society [1], SUI refers to the involuntary release of urine during physical activities, coughing, or sneezing. UUI is characterized by the involuntary loss of urine with urgency. MUI is a condition in which there is involuntary urine leakage associated with urgency, as well as with physical activities, coughing, or sneezing. UI is a prevalent public health issue that has a significant impact on the life quality of numerous populations. Moreover, the condition imposes a substantial economic burden on communities and society [7].

Metabolic syndrome (MetS) is a group of common and complex clinical diseases, including insulin resistance, obesity, hypertension, and dyslipidemia [8]. Insulin resistance (IR) is defined as a physiological status in which insulin-targeting tissues display reduced responsiveness to elevated physiological insulin levels. IR as a crucial component of MetS plays a key role in the description of the pathophysiology of MetS [9, 10]. Moreover, IR is closely connected with various disorders, including type 2 diabetes, atherosclerosis, hypertension, and polycystic ovarian syndrome [11]. The metabolic score for IR (METS-IR) index was a novel score for assessing IR proposed by Bello-Chavolla et al. and it has been shown that METS-IR index has a higher diagnostic effect compared to several non-insulin-based indexes including the TyG index and the TG/HDL ratio [12].

Former studies demonstrated that females with MetS had a higher risk of SUI compared to those without MetS [13]. Yoon et al. showed that IR evaluated by HOMA-IR might be a potential risk factor of UI in postmenopausal non-diabetic females [14]. However, the relationship between METS-IR index and UI has not been explored and reported yet. The current study was conducted to investigate whether there was an association between METS-IR index with UI by using numerous samples of US adults collected from the National Health and Nutrition Examination Survey (NHANES) ranging from 2001 to 2018.

## Materials and methods

### Study description and population

The used data were derived from the National Health and Nutrition Examination Survey (NHANES), a major population-based program carried out by the Centers for Disease Control (CDC) and Prevention’s National Center for Health Statistics (NCHS), aimed at evaluating the condition of health and nutrition among the population of the United State. The combination of interviews and physical examinations is a unique feature of the survey. The content of interviews includes demographic, socioeconomic, dietary, and health-related inquiries. Meanwhile, the examination elements consist of medical, dental, physiological assessments, and laboratory tests, all conducted by trained medical professionals. By utilizing a complex stratified multistage probability design, NHANES collected a nationally representative sample of the noninstitutionalized civilian U.S. population. More detailed information is available at https://www.cdc.gov/nchs/nhanes/index.htm.

**Fig. 1:**
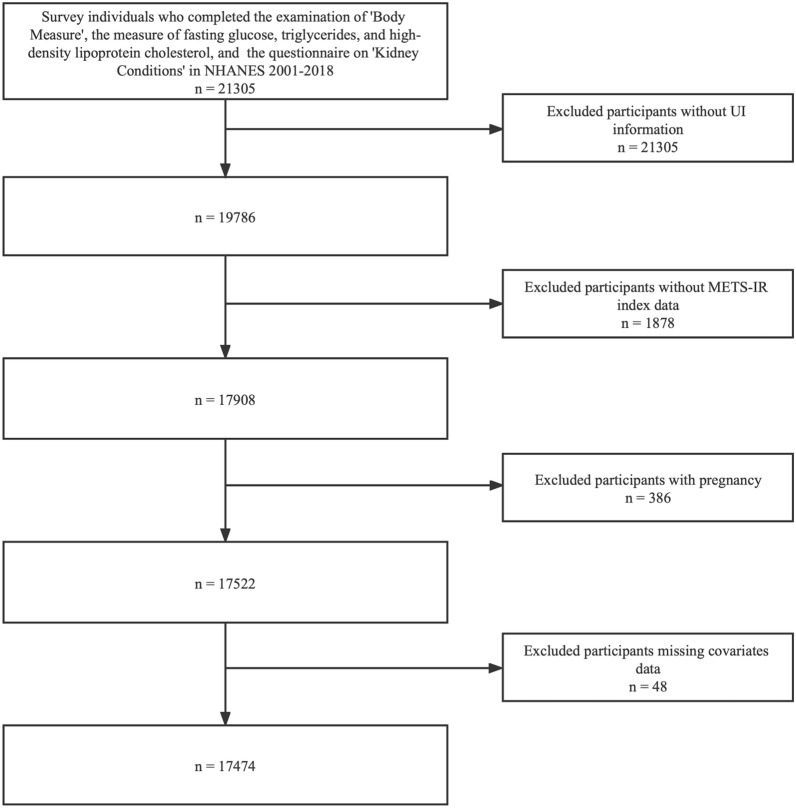
Flow chart of sample selection process

In the current study, the analyzed data were obtained from nine cycles (2001–2018) in the NHANES database. A total of 21,305 participants were enrolled at first. We excluded survey individuals with pregnancy (n = 386), missing METS-IR index data (n = 1878), incomplete UI data (n = 1519), and missing covariate information (n = 48). Ultimately, 17,474 individuals participated in this study (Fig. 1).

### Measurement of METS-IR index

In this study, METS-IR index was set as an exposure variable. METS-IR = Ln[(2 × fasting glucose (mg/dL)) + fasting triglycerides (mg/dL)] × body mass index (kg/m^2^))/[Ln (high-density lipoprotein cholesterol (mg/dL))] [12]. Fasting glucose and triglycerides were enzymatically measured using an automated biochemical analyzer. Specifically, the Roche Cobas 6000 chemistry analyzer and the Roche Modular P were employed for the determination of serum triglyceride concentrations. Body mass index was calculated as weight (kg) divided by the square of height (m), and the data of body weight and height were available in the Examination Data ‘Body Measure’.

### Assessment of UI

Two questions in Questionnaire Data “Kidney Condition” were used to evaluate the occurrence of UI in NHANES database. Participants defined as stress UI (SUI) answered “yes” to the question “During the past 12 months, have you leaked or lost control of even a small amount of urine with an activity like coughing, lifting, or exercise?”. Survey individuals were designed as urgency UI (UUI) if they answered “yes” to the question “During the past 12 months, have you leaked or lost control of even a small amount of urine with an urge or pressure to urinate and you couldn’t get to the toilet fast enough?”. Moreover, if participants responded “yes” to both the above questions, they were set as mixed UI (MUI).

### Covariates of interest

In the current study, covariates of interest included gender, age, race/ethnicity, education level, marital status, the family poverty income ratio (PIR), smoking status, alcohol intake, physical activity (vigorous/moderate), diabetes, hypertension, and high cholesterol. To prevent the reduction of the large sample size in our study, numerous missing covariates for the family PIR (n = 1432) and alcohol intaking (n = 239) were set as missing value categories which were designed as dummy variables in regression models. Survey participants who reported having smoked at least 100 cigarettes over the course of their lifetime and currently smoked every day or some days at the time of the interview were classified as current smokers. Participants who reported having smoked at least 100 cigarettes during their lifetime but were not currently smoking at the time of the questionnaire were designed as former smokers. In addition, to classify survey individuals as nonsmokers, it was determined that those who reported smoking less than 100 cigarettes during their lifetime would be included in this category. Participants were divided into two groups: drinkers (those who consumed at least 12 alcoholic drinks per year) and nondrinkers. Survey subjects who had been diagnosed with diabetes by doctors prior to the interview or had a fasting plasma glucose level ≥ 126 mg/dL were classified as having diabetes. Participants were categorized as having hypertension if they had been diagnosed with the condition by doctors, or were taking medication for hypertension, or had a systolic blood pressure level ≥ 140 mmHg, or had a diastolic blood pressure level ≥ 90 mmHg. If participants were informed by doctors that their cholesterol level was high or were taking medication for hypercholesterolemia, or their total cholesterol value was ≥ 240 mg/dL, they were set as having high cholesterol.

### Statistical analysis

In this study, the sample weights, stratifications, and clustering incorporated in the NHANES study were implemented across all statistical analyses for considering the complex, multistage sampling design employed to select a representative sample of noninstitutionalized civilian U.S. population. Continuous variables were denoted as weighted mean and standard error (SE), and categorical variables were expressed as weighted proportions. In order to compare the baseline characteristics among 4 groups classified by METS-IR index quartiles, a survey-weighted linear regression (continuous variables) and a survey-weighted Chi-square test (categorical variables) were employed.

Multivariable logistic regression models were utilized to explore the relationship between METS-IR index and three types of UI. In Model 1, no covariates were adjusted; Model 2 was adjusted for gender, age, and race; Model 3 was adjusted for gender, age, race/ethnicity, education level, marital status, the family PIR, smoking status, alcohol intake, vigorous activity, moderate activity, diabetes, hypertension, and high cholesterol. Subgroup analysis was conducted to investigate the association between METS-IR index and three types of UI in different stratifications. The employment of smooth curve fitting and generalized additive models allowed for the examination of whether the independent variable was segmented into distinct intervals, thereby assessing the non-linear association between the independent variable and UI. Statistical significance was defined as a two-sided p-value of < 0.05. In the current study, we used EmpowerStats (http://www.empowerstats.com, X&Y Solutions, Inc.) and statistical software packages R (http://www.R-project.org; The R Foundation) in all statistical analyses.

## Results

### Participant characteristics

The detailed baseline characteristics were demonstrated in Table 1. This study involved a sample of 17,474 participants (49.76% male and 50.24% female, based on weighted proportions) with an average age (SE) of 47.85 (0.25) years. Of the study subjects, 23.76% presented with a self-reported history of SUI, 20.05% reported a prior occurrence of UUI, and 9.59% complained of MUI. For quartiles 1–4, the range of METS-IR index was categorized as 17.14–34.39, 34.39–41.56, 41.56–50.19, and 50.19–193.33, respectively. Survey individuals in the higher quartile of METS-IR index had an increased likelihood of all types of UI (p < 0.001).

**Table 1:** Baseline characteristics of participants by the METS-IR index quartiles, weighted

	Total	Q1 (17.14–34.39)	Q2 (34.39–41.56)	Q3 (41.56–50.19)	Q4 (50.19–193.33)	P-value
Participants (n)	17,474	4369	4368	4368	4369	
Age (year), mean (SE)	47.85 (0.25)	44.88 (0.44)	48.95 (0.39)	49.36 (0.36)	48.53 (0.33)	< 0.001
Age (%)						< 0.001
< 50	54.30	60.91	51.77	50.82	53.00	
≥ 50	45.70	39.09	48.23	49.18	47.00	
Gender (%)						< 0.001
Male	49.76	37.29	53.59	56.97	52.57	
Female	50.24	62.71	46.41	43.03	47.43	
Race/ethnicity (%)						< 0.001
Mexican American	8.23	5.23	7.98	10.18	9.87	
Other Hispanic	5.40	4.50	5.58	6.37	5.27	
Non-Hispanic White	69.43	71.90	69.53	67.70	68.33	
Non-Hispanic Black	10.24	9.15	9.86	10.23	11.83	
Other race	6.69	9.22	7.06	5.52	4.70	
Education level (%)						< 0.001
Less than high school	16.44	12.96	16.90	17.83	18.45	
High school or GED	23.93	20.78	23.22	26.40	25.70	
Above high school	59.63	66.26	59.89	55.77	55.85	
Marital status (%)						< 0.001
Living alone	35.64	39.44	36.66	32.02	33.97	
Married or living with partner	64.36	60.56	63.34	67.98	66.03	
Family PIR (%)						< 0.001
≤ 1.3	19.17	17.83	17.36	19.39	22.25	
> 1.3 and ≤ 3.5	34.23	31.26	34.84	35.36	35.78	
> 3.5	40.21	43.85	41.78	38.84	35.97	
Unclear	6.39	7.05	6.01	6.41	6.00	
Smoking status (%)						< 0.001
Current smokers	20.86	23.11	21.51	19.41	19.16	
Former smokers	25.92	21.62	24.94	28.49	29.13	
Nonsmokers	53.22	55.27	53.55	52.10	51.71	
Alcohol intaking (%)						< 0.001
Nondrinkers	27.12	24.83	24.87	28.30	30.77	
Drinkers	71.48	73.86	73.76	70.36	67.65	
Unclear	1.40	1.32	1.37	1.35	1.58	
Vigorous activity (%)						0.003
No	74.25	74.35	73.58	72.24	76.81	
Yes	25.75	25.65	26.42	27.76	23.19	
Moderate activity (%)						0.266
No	54.51	53.34	54.70	54.08	56.03	
Yes	45.49	46.66	45.30	45.92	43.97	
Diabetes (%)						< 0.001
No	86.79	96.81	92.12	85.61	71.54	
Yes	13.21	3.19	7.88	14.39	28.46	
Hypertension (%)						< 0.001
No	61.76	76.91	65.55	58.44	44.49	
Yes	38.24	23.09	34.45	41.56	55.51	
High cholesterol (%)						< 0.001
No	60.03	71.69	59.46	55.91	51.77	
Yes	39.97	28.31	40.54	44.09	48.23	
SUI						< 0.001
No	76.24	78.31	77.75	77.90	70.79	
Yes	23.76	21.69	22.25	22.10	29.21	
UUI						< 0.001
No	79.95	83.33	80.89	80.21	75.01	
Yes	20.05	16.67	19.11	19.79	24.99	
MUI						< 0.001
No	90.41	92.96	91.18	90.18	87.03	
Yes	9.59	7.04	8.82	9.82	12.97	

*Q1–Q4* quartile 1-quartile 4, *SE* standard error, *METS-IR index* metabolic score for insulin resistance index, *GED* general educational development, *Family PIR* family poverty income ratio, *SUI* stressed urinary incontinence, *UUI* urgency urinary incontinence, *MUI* mixed urinary incontinence

### Relationship between METS-IR index and UI

**Fig. 2:**
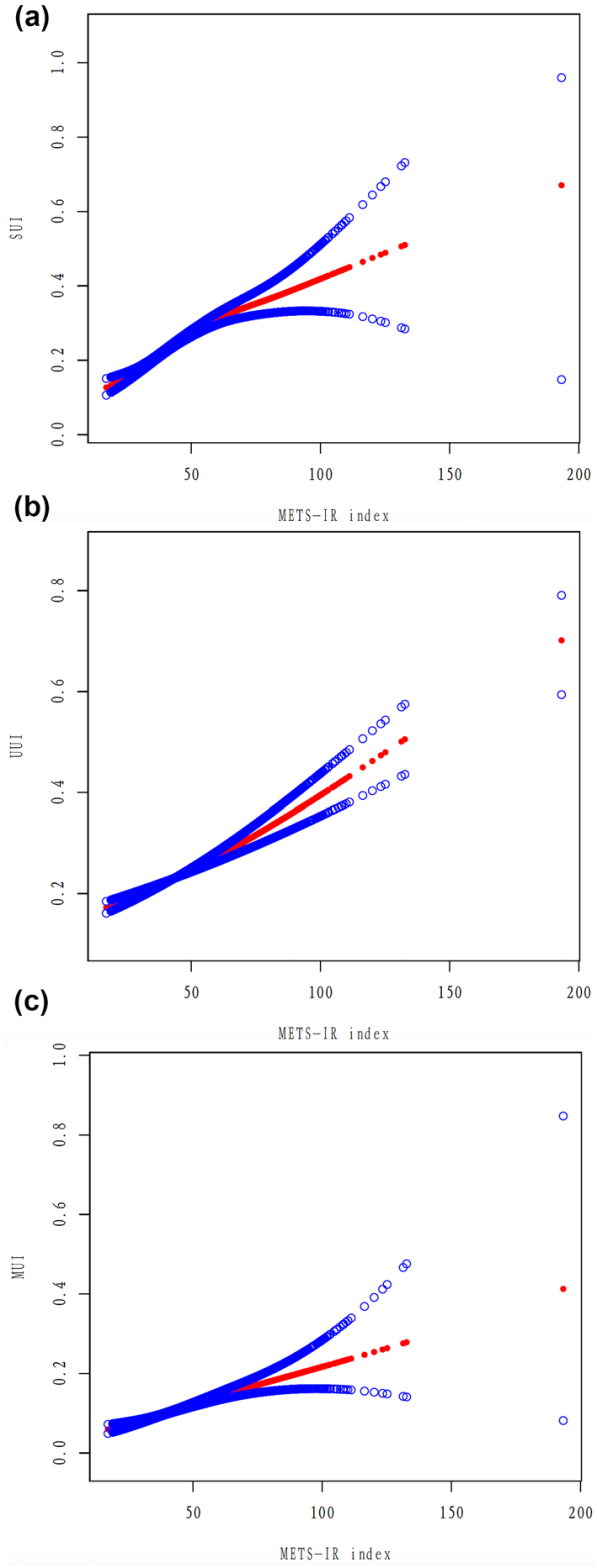
Smooth curve fitting for the relationship between METS-IR index and three types of UI. (**a**), (**b**), and (**c**) represents the linear associations between METS-IR index and SUI, UUI, and MUI, respectively. The area between two blue dotted line is on behalf of a 95% CI. The red dotted line suggests the positive linear relationship between METS-IR index and three types of UI

The association between METS-IR index and UI was evaluated by weighted multivariable logistic regression models in crude (Model 1), minimally (Model 2), and fully adjusted models (Model 3). Our results demonstrated that an elevated METS-IR index was positively related to the higher likelihood of three types of UI. In model 3, the risk of UI increased with each incremental unit in METS-IR index [SUI: odds ratio (OR) = 1.023, 95% confidence interval (95% CI) 1.019–1.027; UUI: OR = 1.015, 95% CI 1.011–1.019; MUI: OR = 1.020, 95% CI 1.016–1.025, all p < 0.001]. Additionally, METS-IR index was transformed from a continuous variable into a categorical variable (Q1–Q4) by quartiles for a sensitivity analysis. In model 3, survey respondents in the highest MEST-IR index quartile (Q4) were at elevated risks for all types of UI compared to those in the lowest quartile (Q1) (SUI: OR = 2.266, 95% CI 1.947–2.637; UUI: OR = 1.534, 95% CI 1.344–1.750; MUI: OR = 2.044, 95% CI 1.707–2.448, all p for trend < 0.001). All detailed information was shown in Table 2. Moreover, the analysis of smooth curves fitting indicated that METS-IR index was positively linearly associated with three types of UI (Fig. 2).

**Table 2:** Association between METS-IR index with urinary incontinence, weighted

SUI	OR (95% CI), P-value		
	Model 1	Model 2	Model 3
Continuous	1.012 (1.008, 1.016), < 0.001	1.027 (1.023, 1.031), < 0.001	1.023 (1.019, 1.027), < 0.001
Categories			
Q1	Reference	Reference	Reference
Q2	1.033 (0.904, 1.182), 0.634	1.511 (1.309, 1.744), < 0.001	1.471 (1.275, 1.696), < 0.001
Q3	1.024 (0.901, 1.165), 0.715	1.685 (1.455, 1.953), < 0.001	1.557 (1.346, 1.802), < 0.001
Q4	1.490 (1.304, 1.702), < 0.001	2.595 (2.247, 2.995), < 0.001	2.266 (1.947, 2.637), < 0.001
P for trend	< 0.001	< 0.001	< 0.001

UUI	OR (95% CI), P-value		
	Model 1	Model 2	Model 3
Continuous	1.017 (1.013, 1.020), < 0.001	1.020 (1.016, 1.024), < 0.001	1.015 (1.011, 1.019), < 0.001
Categories			
Q1	Reference	Reference	Reference
Q2	1.181 (1.032, 1.351), 0.017	1.246 (1.079, 1.439), 0.003	1.191 (1.027, 1.382), 0.021
Q3	1.234 (1.070, 1.422), 0.004	1.328 (1.132, 1.558), < 0.001	1.212 (1.023, 1.437), 0.027
Q4	1.665 (1.469, 1.888), < 0.001	1.829 (1.610, 2.078), < 0.001	1.534 (1.344, 1.750), < 0.001
P for trend	< 0.001	< 0.001	< 0.001

MUI	OR (95% CI), P-value		
	Model 1	Model 2	Model 3
Continuous	1.020 (1.016, 1.024), < 0.001	1.027 (1.022, 1.031), < 0.001	1.020 (1.016, 1.025), < 0.001
Categories			
Q1	Reference	Reference	Reference
Q2	1.278 (1.054, 1.549), 0.014	1.552 (1.276, 1.887), < 0.001	1.442 (1.182, 1.759), < 0.001
Q3	1.438 (1.197, 1.728), < 0.001	1.887 (1.553, 2.291), < 0.001	1.633 (1.328, 2.009), < 0.001
Q4	1.968 (1.668, 2.322), < 0.001	2.584 (2.174, 3.072), < 0.001	2.044 (1.707, 2.448), < 0.001
P for trend	< 0.001	< 0.001	< 0.001

*OR* odds ratio, *95% CI* 95% confidence interval

Model 1: unadjusted

Model 2: adjusted for gender, age, and race/ethnicity

Model 3: adjusted for gender, age, race/ethnicity, education level, marital status, the family poverty income ratio, smoking status, alcohol intaking, vigorous activity, moderate activity, diabetes, hypertension, and high cholesterol

### Subgroup analysis

**Fig. 3:**
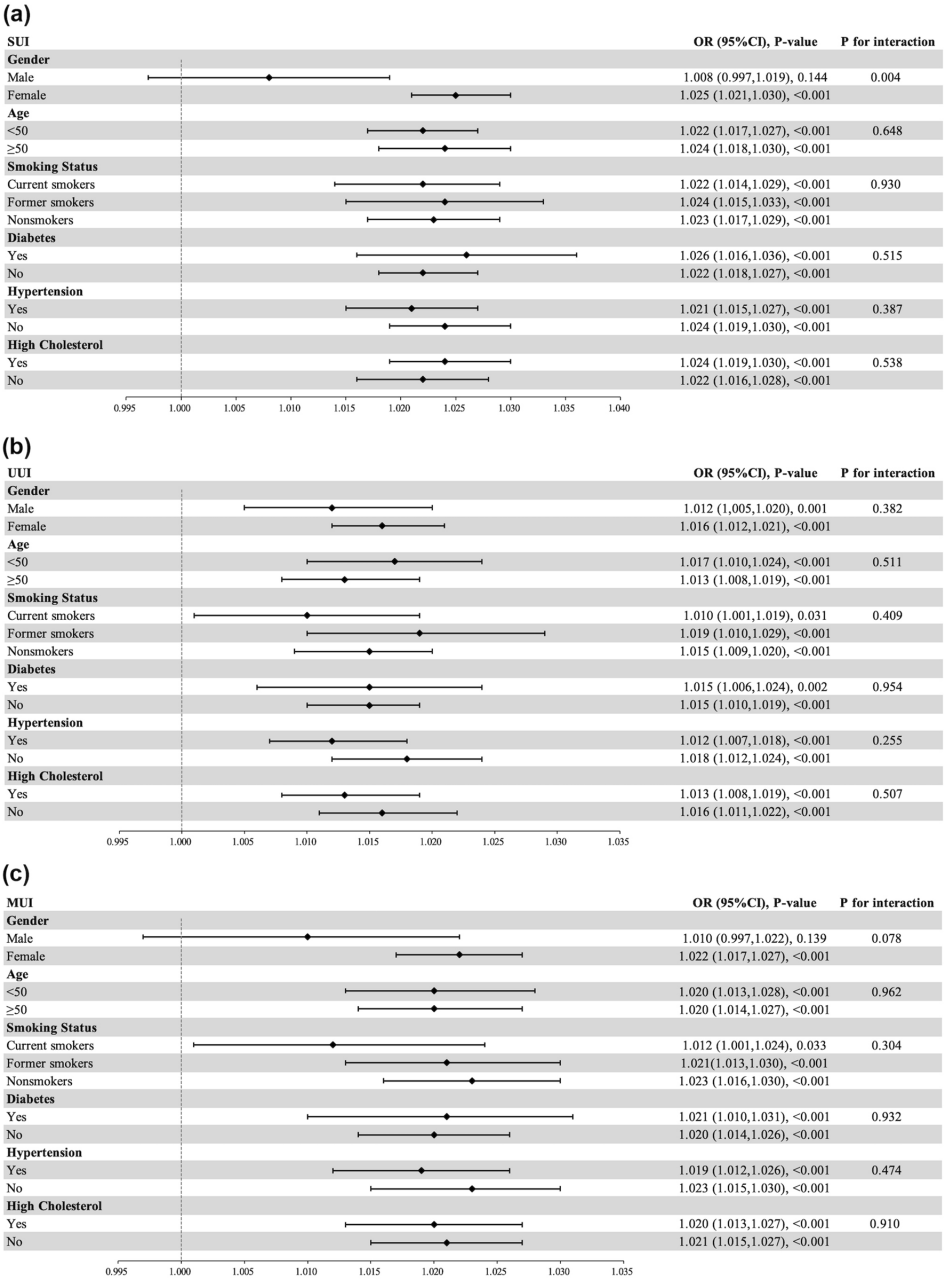
Subgroup analysis for the association between METS-IR index and three types of UI. (**a**), (**b**), and (**c**) represents the results of subgroup analyses for the relationships between METS-IR index and SUI, UUI, and MUI in different stratifications, respectively. All stratified factors include gender, age, race/ethnicity, education level, marital status, the family poverty income ratio, smoking status, alcohol intaking, vigorous activity, moderate activity, diabetes, hypertension, and high cholesterol, except the stratified factor itself

Subgroup analysis was conducted to examine whether the association between METS-IR index and UI was stable in different stratifications. As shown in Fig. 3, the stratified factors included gender, age, smoking status, diabetes, hypertension, and high cholesterol. In Fig. 3a, gender potentially had an impact on the association between METS-IR index and SUI. Compared to males a stronger association of METS-IR index with SUI was observed in female participants (p for interaction < 0.05). In addition, tests for interaction were not significant in all stratifications in UUI and MUI (Fig. 3b, c, respectively) (all p for interaction > 0.05). More detailed information was demonstrated in Fig. 3.

## Discussion

In this study, a large and representative sample was obtained from the NHANES database 2001–2018 to assess the association between METS-IR index and UI in the US population. The results suggested that an elevated METS-IR index was related to a higher likelihood of UI, including SUI, UUI, and MUI. In addition, we converted METS-IR index from a continuous variable to a categorical variable by quartiles of METS-IR index and discovered that the positive relationship between METS-IR index and UI was still stable. Smooth curve fitting showed the linearly positive connection between METS-IR index and UI. Moreover, subgroup analysis demonstrated that a stronger relationship between METS-IR index and SUI was observed in women than in men.

IR is a core part of MetS, evaluated by various methods and indexes. The euglycemic–hyperinsulinemic clamp (EHC) is the gold standard for assessing IR. However, it is limited in clinical applications because it is time-consuming and expensive [15]. The homeostatic model assessment for IR (HOMA-IR) is a widely used index for IR based on fasting insulin [16], while the measurement of fasting insulin is not routine progress and is expensive in some undeveloped countries. METS-IR index as a novel indirect score for IR not based on the test for fasting insulin was first reported in 2018 and was calculated by several non-insulin fasting laboratory indicators and an anthropometric parameter that were easily measured. In addition, METS-IR index demonstrated that it had a good predictive effect on the risk of type 2 diabetes and could assess the cardiometabolic risk [12]. Moreover, various studies showed that METS-IR index was closely linked to various disorders such as hypertension, coronary artery calcification, and diabetes [17–19]. However, the association between METS-IR index and UI has not been investigated. This study innovatively investigated the value of METS-IR index at the onset of UI. UI is a disease with a significant burden of disease, especially for women, with more than 17% of women over the age of 20 suffering from UI [20]. However, there are still few indicators that can effectively predict the occurrence of UI, and the risk factors for UI have not yet been fully investigated. As a simple, reliable, and reproducible predictor, METS-IR index plays an important role in predicting the occurrence of UI. The application of METS-IR index to predict UI in clinical practice will be beneficial for health guidance and early intervention in high-risk populations and will reduce the risk of UI to some extent. Additionally, the relationship between IR and UI was not very clear. This study has initially explored the correlation, providing the clue for further in-depth research.

IR is closely related to various pathophysiologic alterations, which may result in the incidence of UI. It has been demonstrated that IR is closely linked to inflammation. The suppression of the anti-inflammatory effects of insulin caused by IR may promote the progress of inflammation. Dandona et al. showed that insulin exerted the obvious anti-inflammatory effect by the downregulation of intranuclear nuclear factor kappaB and the upregulation of IkappaB in mononuclear cells. In addition, insulin reduced the generation of reactive oxygen species and p47^phox^ in mononuclear cells and suppressed the production of plasma soluble intercellular adhesion molecule-1 (ICAM-1) and monocyte chemoattractant protein-1 (MCP-1) [21]. Furthermore, the high expression of inflammatory mediators such as tumor necrosis factor-α (TNF-α), interleukin-6 (IL-6), C-reactive protein (CRP), and MCP-1 has an impact on the formation of IR [22, 23]. Previous studies have explored the impact of inflammation on UI. Shinohara et al. demonstrated that TNF-α suppressed the myogenic differentiation of human urethral rhabdosphincter cells, suggesting that TNF-α may be a risk factor of SUI in the elderly [24]. Additionally, inflammation also plays a role in overactive bladder (OAB) associated with UI. Chung et al. discovered that participants with OAB had a significantly higher CRP level compared to those without OAB [25]. Moreover, the inflammation induced by MetS may be a crucial factor in the manifestation of lower urinary tract symptoms (LUTS) in males and has been suggested as a potential mechanism that links MetS with LUTS [26]. Former studies demonstrated that IR may be a key factor in the loss of muscle mass and had a potential impact on the formation of sarcopenia [27, 28]. Pelvic floor muscles are key structures for the maintenance of urinary continence. Pelvic floor muscle training is an effective prevention and treatment for UI in females [29, 30]. It has been studied that sarcopenia is related to UI [31]. Oxidative stress is a typical and important mechanism related to IR. Tinahones et al. indicated that a reduced activity of superoxide dismutase (the enzyme eliminating the superoxide anions generation) was observed in obese persons with greater IR, resulting in a higher level of superoxide anions [32]. The previous study suggested that nonobese children with IR had a lower antioxidant status [33]. In addition, the use of antioxidant agents could ameliorate the status of IR [34]. The effect of oxidative stress on UI has been investigated. Nocchi et al. showed that oxidative stress compromised the function of the urothelium in mice via TRPM8 [35]. It is well known that IR is highly related to the risk of diabetes. Diabetes and UI as the common chronic conditions have the potential association. Phelan et al. concluded multiple clinical trials exploring the relationship between diabetes and UI and suggested that the prevalence of UI was increased in females with type 2 diabetes [36]. The specific mechanisms that connect IR and UI and whether IR is an independent risk factor for UI need to be explored by more investigations.

This study used the collected data from the NHANES database and explored the association between METS-IR index and UI in a large and representative sample in the United States. The sampling design and weighting were utilized in the statistical analyses for representing the general adults in the US. However, there are several limitations in the current study. First, this is a cross-sectional study, thus the causal relationship between METS-IR index and UI cannot be investigated. Additionally, constrained by the questionnaire design for UI in the NHANES database, the symptoms and history of three types of UI were self-reported by participants through responding to the interviews, probably resulting in an underestimation of the actual number of UI individuals. Due to variations in how participants interpret questions, differences in educational levels, and other factors, this kind of self-reporting questionnaire may lead to differences in participants’ perception of their own health conditions and introduce bias to some extent. The database used the binary response format questionnaire to assess patients with UI, which may increase the bias due to subjective factors to some extent and neglect to assess the extent of UI in the population. The self-reporting and binary assessment survey model should indeed be the focus of attention. Finally, it is important to note that the NHANES database only provided data on the United States population. Therefore, further studies are needed to corroborate the linkage between METS-IR index and UI in various national populations.

## Conclusion

To the best of our knowledge, this is the first cross-sectional study to examine the association between METS-IR index and UI in the adult population of the United States. In this study, a higher METS-IR index was related to an elevated likelihood of three types of UI (SUI, UUI, and MUI). After METS-IR index was divided into 4 groups by quartiles, the positive connections were still stable. However, more research is needed to validate our findings.

## Data Availability

Publicly available datasets were analyzed in the present study. All detailed data can be found here: www.cdc.gov/nchs/nhanes/.
